# Circulating Levels of Hormones, Lipids, and Immune Mediators in Post-Traumatic Stress Disorder – A 3-Month Follow-Up Study

**DOI:** 10.3389/fpsyt.2015.00049

**Published:** 2015-04-14

**Authors:** Mladen Jergović, Krešo Bendelja, Ana Savić Mlakar, Valerija Vojvoda, Neda Aberle, Tanja Jovanovic, Sabina Rabatić, Ante Sabioncello, Anđelko Vidović

**Affiliations:** ^1^Centre for Research and Knowledge Transfer in Biotechnology, University of Zagreb, Zagreb, Croatia; ^2^Department for Cellular Immunology, Institute of Immunology, Zagreb, Croatia; ^3^General Hospital “Dr. Josip Benčević”, Slavonski Brod, Croatia; ^4^Department of Psychiatry and Behavioral Sciences, Emory University, Atlanta, GA, USA; ^5^Department of Psychiatry, University Hospital Dubrava, Zagreb, Croatia

**Keywords:** post-traumatic stress disorder, veterans, biological markers, cholesterol, cytokines, cell adhesion molecules, nerve growth factor, leptin

## Abstract

A number of peripheral blood analytes have been proposed as potential biomarkers of post-traumatic stress disorder (PTSD). Few studies have investigated whether observed changes in biomarkers persist over time. The aim of this study was to investigate the association of combat-related chronic PTSD with a wide array of putative PTSD biomarkers and to determine reliability of the measurements, i.e., correlations over time. Croatian combat veterans with chronic PTSD (*n* = 69) and age-matched healthy controls (*n* = 32), all men, were assessed at two time points separated by 3 months. Serum levels of lipids, cortisol, dehydroepiandrosterone-sulfate (DHEA-S), prolactin, and C-reactive protein were determined. Multiplex assay was used for the simultaneous assessment of 13 analytes in sera: cytokines [interferon-γ, interleukin (IL)-1β, IL-2, IL-4, IL-6, TNF-α], adhesion molecules (sPECAM-1, sICAM-1), chemokines (IL-8 and MIP-1α), sCD40L, nerve growth factor, and leptin. Group differences and changes over time were tested by parametric or non-parametric tests, including repeated measures analysis of covariance. Reliability estimates [intraclass correlation coefficient (ICC) and kappa] were also calculated. Robust associations of PTSD with higher levels of DHEA-S [*F*(1,75) = 8.14, *p* = 0.006)] and lower levels of prolactin [*F*(1,75) = 5.40, *p* = 0.023] were found. Measurements showed good to excellent reproducibility (DHEA-S, ICC = 0.50; prolactin, ICC = 0.79). Serum lipids did not differ between groups but significant increase of LDL-C after 3 months was observed in the PTSD group (*t* = 6.87, *p* < 0.001). IL-8 was lower in the PTSD group (*t* = 4.37, *p* < 0.001) but assessments showed poor reproducibility (ICC = −0.08). Stable DHEA-S and prolactin changes highlight their potential to be reliable markers of PTSD. Change in lipid profiles after 3 months suggests that PTSD patients may be more prone to hyperlipidemia. High intra-individual variability in some variables emphasizes the importance of longitudinal studies in investigations of PTSD biomarkers.

## Introduction

Long-term mental and physical health consequences of trauma place a significant burden on healthcare systems, especially in areas with a recent history of conflict where the majority of the population has been affected by war (1, 2). Post-traumatic stress disorder (PTSD) has been found to be the most frequent mental disorder 5–15 years after the war in the Balkans (3). Moreover, PTSD has been associated with various somatic comorbidities with particularly strong evidence for higher prevalence of cardiovascular (4–6) and autoimmune (7–9) diseases. Various metabolic, hormonal, and immune-related changes that could lead to somatic diseases have been observed in PTSD. In the majority of studies to date, PTSD patients exhibited unfavorable lipid profiles, either alone (10–15) or as a part of metabolic syndrome (16, 17), which reflects increased cardiovascular disease (CVD) risk. There is also substantial evidence for dysregulation of the hypothalamic–pituitary–adrenal (HPA) axis with decreased levels of blood and urinary cortisol and enhanced sensitivity of the HPA axis to negative feedback (18). These alterations could influence lipid metabolism (19) and contribute to immune system activation leading to peripheral inflammation reflected by elevated circulating levels of cytokines and acute phase reactant C-reactive protein (CRP) (20). Given the complex neuroendocrine–immune interactions observed in PTSD, researchers have utilized assays that allow for simultaneous detection of multiple analytes in a limited amount of sample and found a broad spectrum of increased circulating peripheral levels of pro-inflammatory cytokines and chemokines (21, 22). Prolactin, as an immunostimulatory hormone (23), may be partly involved in the processes leading to immune activation in PTSD (24). Neurosteroid dehydroepiandrosterone (DHEA) and its sulfated ester form dehydroepiandrosterone-sulfate (DHEA-S) are other components of the HPA axis that may be altered in PTSD (25) and contribute to immune activation through their immunostimulatory and anti-glucocorticoid effects (26). A substantial number of reports showed elevated DHEA and/or DHEA-S concentrations in PTSD patients (27–33).

However, results from many studies are not consistent with the above mentioned findings in PTSD. For example, studies have reported serum lipids as unchanged (34, 35), and no change or elevation in basal cortisol levels (18, 36, 37). Prolactin levels were found to be decreased or unchanged (38–41), as well as DHEA(-S) levels (42–44) in addition to reports of no change in basal circulating levels of multiple cytokines (45). As for other potential biomarkers of PTSD, the reasons for these inconsistencies are complex and multifactorial (46) and as a result, no valid and reliable single PTSD biomarker has been identified to date (47).

While there are a growing number of prospective studies investigating PTSD risk/resilience markers (48, 49), studies of disease markers have typically been cross-sectional. Biological markers show substantial variability with great overlap between PTSD and non-PTSD groups, so large sample sizes are needed to obtain enough statistical power to confirm differences. Measurements are seldom repeated during the course of the disease to assess how stable the observed changes are over time.

The aim of this study was to investigate the association of combat-related chronic PTSD with the serum levels of lipids, stress-related hormones (cortisol, DHEA-S, prolactin), CRP, cytokines and other immune-related soluble molecules, nerve growth factor (NGF), and leptin. The second aim was to determine whether the observed changes remained consistent after 3 months during the course of disease and measurement reliability. We hypothesized that biological changes associated with PTSD, i.e., possible PTSD biomarkers, would be inert to repetitive testing during the course of chronic PTSD.

## Material and Methods

### Subjects

Ninety-eight PTSD patients were recruited among outpatients at the “Dr. Josip Benčević” General Hospital, Slavonski Brod, Croatia. The patients were male combat veterans who were severely traumatized during the war in Croatia (1991–1995) and have undergone several forms of psychiatric treatment since the war ended. Before inclusion in the study, patients have been continuously treated in an outpatient program for at least 10 years. They were at least once treated in a day hospital program and/or inpatient program. The treatment included supportive psychotherapy and/or cognitive-behavioral therapy as well as various sociotherapeutic interventions. During this period, every patient received multiple medication trials. Considering all this, the patients could be regarded as treatment-resistant (50). During the recruitment, all patients met the ICD-10 (51) PTSD criteria (the official classification in Croatian psychiatric practice) and they were treated in an outpatient program, which included regular appointments with psychiatrist and medications. Due to the ethical considerations, medication washout could not be performed. The medications prescribed were recorded based on patients’ last visit to psychiatrist before assessments. The type of medication did not change during the study period. Patients were asked to participate in the study during the regular appointment in the hospital or by telephone call. The study procedures were fully explained to them and they were instructed to restrict from food intake at least 12 h before the blood draw, which was scheduled at 7–9 a.m. on the day of the assessments. They were also asked to inform their friends and relatives who were not treated for PTSD about possible participation in the study. We were able to recruit 33 control subjects among patients’ and researchers’ acquaintances and hospital workers. They were age-matched men who had been civilian residents of the same (war-affected) area during the war and were never treated for PTSD. The Ethic Committee of the hospital approved the study and written informed consent was obtained from all subjects on the day of the first assessment. A subset of blood samples randomly selected from participants in this study was used to examine the phenotype of regulatory T cells in PTSD patients and this data have recently been published (52).

The first assessment (time 1) was performed on November 11 2009. At the day of the assessment, all study participants were examined by an experienced psychiatrist and medical history data relevant to the purpose of this study were recorded. After measuring blood pressure and collecting the blood sample, the subjects were asked to complete rating scales for PTSD, depression, and anxiety symptoms. The diagnosis was confirmed if a participant fulfilled criteria for PTSD by rating 17 diagnostic items of the 43-item version of the Los Angeles Symptom Checklist (LASC) (53). This instrument proved to be highly a reliable self-report symptom checklist that includes the DSM-III-R (54) criteria for PTSD as a subset [coefficient alpha of 0.94 for the 17 items specifically assessing PTSD symptoms (55)] and it was used to quantify re-experiencing, avoidance, and arousal symptoms of PTSD. Respondents rated the extent to which specific symptoms were a problem for them, using a 5-point Likert-type scale ranging from 0 (no problem) to 4 (extreme problem). For a diagnosis of PTSD, the respondent must rate (with a rating of two or higher) at least one item assessing re-experiencing (represented by three items), three items (out of six items) related to avoidance and numbing, and two items reflecting increased arousal (represented by eight items). If a respondent endorses two of the three criteria, a partial PTSD diagnosis may be considered. Depression symptoms were assessed with the Beck Depression Inventory (BDI) (56) and anxiety level (state and trait) was determined by the Spielberger State-Trait Anxiety Inventory (STAI) (57). Demographic data, smoking, and alcohol use were assessed by a custom questionnaire. Current smokers were participants who reported smoking cigarettes every day (daily) or some days (non-daily). Participants who were former smokers or never smoked a cigarette were considered non-smokers. Frequency of alcohol use during the past 12 months was determined on the basis of following response categories (separately for wine/beer and spirits): (1) every day, (2) 3–5 times/week, (3) once a week, (4) less than once a month, (5) never. Since all participants who drank responded with categories 2 and 3, we decided to merge these categories and report alcohol consumption as “at least one drink per week.”

Healthy controls did neither report experiences that would classify as criterion A (traumatic event) for PTSD diagnosis nor did they meet lifetime or current criteria for any psychiatric disorder and had no symptoms or signs of current psychiatric disease. Exclusion criteria for both groups were substance abuse, symptoms or signs of acute or chronic physical illnesses, including infectious, allergic, or endocrine disorders, and the use of glucocorticoids.

Five patients were excluded because they did not fulfill criteria for PTSD diagnosis. They reported traumatic event(s) (as opposed to controls) but did not satisfy a single PTSD criterion according to LASC 17-item PTSD index. Fifteen patients were excluded because of chronic illnesses: asthma (*n* = 1), diabetes (*n* = 3), multiple sclerosis (*n* = 2), chronic pancreatitis (*n* = 1), Parkinson disease (*n* = 1), psoriasis (*n* = 2), ulcerative colitis (*n* = 1), and cancer (*n* = 4). Another eight patients and one control were excluded because they reported at least one general symptom of current somatic disease (e.g., fever or fatigue) and had CRP >10 mg/L indicating acute inflammation or infection. They were asked to repeat blood tests after a week and CRP levels went down to normal. One patient was excluded because of age (>65 years). This yielded a total of 29 patients and 1 control subject who were excluded from the study.

The second assessment (time 2) was performed on February 8, 2010 and all procedures performed at the first assessment were repeated by the same group of professionals to minimize sources of variability. Six PTSD patients and five control subjects did not show up at the follow-up.

### Blood sampling and assays

Blood samples were taken by venipuncture from all participants on the same day between 7 and 9 a.m. at both assessments. Blood was collected into 10-mL Vacutainer tubes (Becton Dickinson Vacutainer System Europe, Grenoble, France) without anticoagulant. One serum tube was sent to the hospital laboratory for immediate determination of serum lipids and CRP levels. Triglycerides, total cholesterol, high-density lipoprotein cholesterol (HDL-C), and low-density lipoprotein cholesterol (LDL-C) were determined on a Beckman Coulter AU680 analyzer (Beckman Coulter, Mishima, Japan) (58). Determination of CRP was performed using a standard latex immunoassay; CRP Vario (Abbott Diagnostics, Lake Forest, CA, USA). Another serum tube was transported to the research laboratory for determination of cortisol, DHEA-S, and prolactin concentrations, as well as concentrations of analytes that were measured by multiplex immunoassay (henceforth termed “multiplex analytes”). Separation of sera in the research laboratory was performed within 2 h of the blood draw. After separation, the sera were stored at −80°C until assayed.

Serum concentrations of cortisol, DHEA-S, and prolactin were determined by enzyme-linked immunosorbent assay (ELISA) kits from the same manufacturer in both assessments (NovaTec Immunodiagnostica GmbH, Dietzenbach, Germany) according to the manufacturer’s instructions. The sensitivity of the cortisol assay was 5 ng/mL, and the intra-assay and inter-assay coefficients of variation (CV) were 7 and 9%, respectively. DHEA-S assay had the sensitivity of 0.045 μg/mL with the intra-assay CV of 4.8% and the inter-assay CV of 8.9%. The sensitivity of the prolactin assay was 1 ng/mL with the intra-assay and inter-assay CVs of 1.4–3.5 and 3.5–5.4%, respectively.

Simultaneous determination of 13 analytes per one serum sample was performed by bead-based multiplex immunoassay for the flow cytometer (FlowCytomix™ Multiple Analyte Detection System, Bender MedSystems GmbH, Vienna, Austria). The assay was customized to include following analytes: cytokines [interferon (IFN)-γ, interleukin (IL)-1β, IL-2, IL-4, IL-6, TNF (tumor necrosis factor)-α], soluble (s) adhesion molecules [sPECAM (platelet endothelial cell adhesion molecule)-1, sICAM (intercellular adhesion molecule)-1], chemokines [macrophage inflammatory protein (MIP)-1α and IL-8], sCD40 ligand (L), NGF, and leptin. Assay sensitivities for each analyte are included in Table 3. Intra- and inter-assay CVs were all below 10% except IL-4, with the documented intra-assay CV of 15% and inter-assay CV being 16.3%. Samples were analyzed with LSR II flow cytometer (BD Biosciences, Mountain View, USA) and official manufacturer’s software (FlowCytomix™ Pro Software, Bender MedSystems GmbH, Vienna, Austria) was used to calculate analyte concentrations.

### Statistical analyses

Distribution normality for all continuous variables was assessed within each group by visual inspection of the data (shape and symmetry of distribution) and by Shapiro–Wilk’s *W*-test.

Participants’ characteristics were compared with independent sample *t*-tests, Fisher’s exact tests for 2 × 2 contingency tables, and Pearson’s chi-square tests for larger contingency tables.

Data for serum lipids, hormones measured by ELISAs, and CRP were available for both groups at both assessments. Since there were 11 dropouts (6 in the PTSD group and 5 in the control group), missing data analysis was performed to test if data were missing at random. Available data at the first assessment were compared between missing and non-missing groups using non-parametric tests (Fisher’s exact test or Pearson’s chi-square test for categorical variables and Mann–Whitney’s test for continuous variables). Another test of randomness was a logistic regression predicting missingness at the second assessment (0 = not missing, 1 = missing) from all other variables at the first assessment. Since all the tests confirmed that there was no difference between missing and non-missing groups, we concluded that data were missing completely at random (MCAR). Therefore, we assumed that estimates would not be biased if we analyzed only available cases. Due to technical reasons, some values at the second assessment were still missing in the PTSD group (two missing values for cholesterol, triglycerides, HDL, CRP, and five missing values of LDL) and in the control group (one missing value for cholesterol, triglycerides, HDL, LDL, and CRP distributed among different cases). These values were imputed with the corresponding value from the data obtained at the first assessment (last observation carried forward method). Variables that were not normally distributed (triglycerides, DHEA-S, prolactin, CRP) were log-transformed. Repeated measures analysis of covariance (RM ANCOVA) was performed to test the effect of time (within-subjects, two levels) and group (between-subjects, two levels). If the interaction effect was significant, *post hoc t*-tests for dependent or independent samples were performed. Possible confounding factors (included in the model as covariates) were chosen based on previous studies and their expected influence of medication on biological variables. These factors included age, smoking (current smoker vs. non-smoker), alcohol use (at least one drink per week vs. never), and medication use (10 binary variables related to prescribed drug type). All variables followed multivariate normal distribution as tested by Box’s test of equality of covariance matrices.

A multiplex assay was performed in all subjects at the first assessment, but due to the lack of resources, analysis was repeated in randomly selected 47 PTSD patients. Using the same approach as described above, missing data analysis confirmed that there was no difference between selected PTSD patients and other patients in variables assessed at the first assessment. Samples with values below the lower limit of detection (LOD) were replaced with a zero for the purpose of continuous data analyses. Seven out of 13 multiplex analytes (sCD40L, sPECAM-1, sICAM-1, Leptin, MIP-1α, IL-8, IL-1β) had concentrations below LOD in <30% of samples and they were analyzed as continuous variables. Other variables (IL-2, TNF-α, IFN-γ, NGF, IL-4, IL-6) were dichotomized (detectable vs. non-detectable) and analyzed as categorical variables. Continuous variables were log-transformed to obtain normal distribution and were compared with *t*-tests for independent and dependent samples. Since IL-1β did not follow a Gaussian distribution even after every commonly used transformation, non-parametric Mann–Whitney’s and Wilcoxon signed-rank tests were used. Categorical variables were compared using Fisher’s exact tests or McNemar’s tests for paired data. To minimize the possibility of type I error because of the multiple comparisons performed, we used the false discovery rate method (FDR) (59). FDR threshold value (*d*) was calculated with significance level (α) set to 0.05 for 26 comparisons related to multiplex analytes. One-way ANCOVA was used to check the influence of confounding factors in variables that were significantly different between groups. To test for associations between confounding factors and other variables, subgroup analyses with non-parametric (Mann–Whitney’s *U* or Fischer’s exact) tests were performed.

The intraclass correlation coefficient (ICC) was calculated for continuous variables to test the reliability (i.e., the ability to differentiate between subjects or group of subjects) of laboratory methods and psychometric tests. To calculate ICC, we used one-way random-effects ANOVA model and the following formula: $\sigma_{B}^{2}/\left(\sigma_{W}^{2}+\sigma_{B}^{2}\right)$; between-subject variance/(within-subject variance + between-subject variance). Although repeated measurements were performed by the same methods and the same raters, they were separated by a relatively long period of time. Therefore, we assumed that measurements were made under changing conditions, which means that the ICC was an estimate of reproducibility (60). According to Fleiss (61), an ICC ≥0.75 indicates excellent reproducibility, 0.4 ≤ ICC < 0.75 indicates fair to good reproducibility, and ICC <0.4 indicates poor reproducibility. Additionally, kappa coefficients were calculated to evaluate agreement between measurements of the multiplex analytes that were represented as binary variables. As suggested by Altman (62), κ < 0.20 represents poor agreement, 0.21–0.40 fair, 0.41–0.60 moderate, 0.61–0.80 good, and 0.81–1.00 a very good agreement.

Correlations between the psychometric test scores and biological variables were performed with Spearman’s rank order correlations. A total of 168 correlations (21 biological × 8 psychometric variables) were performed in each time point and between time points. The FDR method was used to account for multiple correlations.

Statistical analyses were performed with Statistica v8 (StatSoft Inc., Tulsa, OK, USA).

## Results

### Participants’ characteristics and psychometric test scores

As seen in Table 1, groups were matched in age and marital status. PTSD patients were less educated with a higher proportion of control subjects having university education. The majority of PTSD patients were retired, as opposed to controls. There were more current smokers in the PTSD group but a higher proportion of control subjects reported alcohol consumption (at least one drink per week). Blood pressure did not differ between groups and did not change over 3 months. As expected, PTSD patients had higher scores in all psychometric tests at the first assessment (Table 1). Although the level of PTSD symptoms that patients reported declined [re-experiencing, 5.4 ± 2.6 (score ± SD), *t* = 9.25, *p* < 0.001 (compared to the first assessment); avoidance, 9.0 ± 5.3, *t* = 7.23, *p* < 0.001; arousal, 13.1 ± 6.3, *t* = 11.14, *p* < 0.001; 17-item PTSD index, 27.5 ± 13.2, *t* = 10.9, *p* < 0.001; 43-item index, 55.0 ± 28.1, *p* < 0.001], they were still higher when compared to control subjects at the second assessment [re-experiencing, 1.1 ± 1.6, *t* = 9.49, *p* < 0.001; avoidance, 3.26 ± 3.9, *t* = 5.73, *p* < 0.001; arousal, 4.9 ± 5.7, *t* = 6.11, *p* < 0.001; 17-item PTSD index, 9.2 ± 10.4, *t* = 6.87, *p* < 0.001; 43-item index, 21.4 ± 21.9, *t* = 6.09, *p* < 0.001]. Fourteen out of 69 patients (20%) did not meet diagnostic criteria for PTSD. Since those patients endorsed two of the three LASC criteria, their condition could be regarded as partial PTSD. Similarly, BDI scores also declined in PTSD patients (12.3 ± 10.2, *t* = 12.08, *p* < 0.001) but remained elevated in comparison to controls at the second assessment (4.7 ± 6.3, *t* = 4.13, *p* < 0.001). The same pattern was also observed for STAI scores (data not shown). The majority of PTSD patients (*n* = 57, 83%) were treated with psychotropic medication: antidepressants (*n* = 47, 68%), anxiolytics (*n* = 49, 71%), hypnotics (*n* = 46, 67%), antipsychotics (*n* = 16, 23%), and mood stabilizers (*n* = 3, 4%). Other drugs that PTSD patients used included non-steroidal anti-inflammatory drugs (*n* = 12, 17%), opioid analgesics (*n* = 2, 3%), hypolipidemics (*n* = 7, 10%), antihypertensives (*n* = 12, 17%), and proton pump inhibitors (*n* = 6, 9%).

**Table 1:** **Participants’ characteristics and psychometric test scores (first assessment)**.

	PTSD (*n* = 69), mean ± SD or *n*(%)	Controls (*n* = 32), mean ± SD, or *n* (%)	Statistical value	*p*-value
Age (years)	47.12 ± 5.92	45.56 ± 7.24	1.14^a^	0.256
Education
Elementary school	12 (17.4)	0 (0)	9.53^b^	0.009
High School	52 (75.4)	25 (78.1)	
University	5 (7.2)	7 (21.9)	
Work status
Unemployed	5 (7.2)	1 (3.1)	76.30^b^	<0.001
Retired	60 (87.0)	1 (3.1)	
Employed	4 (5.8)	30 (93.8)	
Marital status
Single/divorced	14 (20.3)	2 (6.2)	Exact test^c^	0.072
Married	55 (79.7)	30 (93.8)	
Current smoker	37 (53.6)	5 (15.6)	Exact test^c^	<0.001
Alcohol use	14 (20.3)	14 (43.8)	Exact test^c^	0.014
Systolic blood pressure (mmHg)	131.5 ± 17.1	132.6 ± 23.1	0.30^a^	0.766
Dyastolic blood pressure (mmHg)	84.3 ± 12.0	85.6 ± 14.7	0.49^a^	0.625
LASC scores
Re-experiencing	8.4 ± 1.8	1.4 ± 1.9	17.54^a^	<0.001
Avoidance	15.0 ± 3.6	4.3 ± 4.3	12.95^a^	<0.001
Arousal	22.6 ± 5.0	5.0 ± 5.2	17.24^a^	<0.001
17-item PTSD index	46.1 ± 8.8	10.7 ± 10.7	17.51^a^	<0.001
43-item full scale index	95.0 ± 21.3	24.4 ± 23.6	14.94^a^	<0.001
BDI score	31.1 ± 9.0	5.3 ± 5.8	14.77^a^	<0.001
STAI-State score	58.4 ± 11.3	37.7 ± 9.9	9.21^a^	<0.001
STAI-Trait score	58.8 ± 6.9	37.5 ± 11.6	12.29^a^	<0.001

*BDI, Beck Depression Inventory; LASC, Los Angeles Symptom Checklist; PTSD, post-traumatic stress disorder; SD, standard deviation; STAI, Spielberger State-Trait Anxiety Inventory*.

*^a^*t*-value: independent sample *t*-tests*.

*^b^χ^2^-value: Pearson’s chi-square tests for larger contingency tables*.

*^c^Exact test: Fisher’s exact tests for 2 × 2 contingency table*.

### Serum lipids, hormones, and CRP

Results of RM ANCOVA analyses controlling for age, smoking, alcohol use, and medication use (10 variables) are shown in Table 2.

**Table 2:** **Serum lipids, hormones, and C-reactive protein (CRP) analyzed by repeated measures analysis of covariance (RM ANCOVA)**.

Variable^a^	Time 1, mean ± SD or median (Q1–Q3)		Time 2, mean ± SD or median (Q1–Q3)		RM ANCOVA effects (d*f* = 1,75)^b^		
	PTSD (*n* = 63)	Controls (*n* = 27)	PTSD (*n* = 63)	Controls (*n* = 27)	Group, *F* (*p*)	Time, *F* (*p*)	Interaction, *F* (*p*)
Serum lipids
Triglycerides (mmol/L)	1.54 (1.27–2.29)	1.89 (1.17–2.95)	2.11 (1.47–2.97)	1.94 (1.40–3.05)	2.64 (0.108)	0.14 (0.707)	0.23 (0.631)
Total cholesterol (mmol/L)	5.69 ± 1.05	5.86 ± 0.73	6.04 ± 1.14	5.98 ± 1.14	0.39 (0.535)	2.32 (0.133)	1.24 (0.269)
LDL-C (mmol/L)	3.26 ± 0.79	3.67 ± 0.97	3.82 ± 0.97	3.76 ± 1.14	0.11 (0.737)	1.48 (0.227)	**5.04 (0.028)**
HDL-C (mmol/L)	1.12 ± 0.18	1.07 ± 0.21	1.10 ± 0.21	1.14 ± 0.21	0.24 (0.629)	0.26 (0.612)	0.13 (0.716)
Serum hormones
Cortisol (ng/mL)	313.89 ± 87.84	296.45 ± 63.02	200.57 ± 71.61	215.12 ± 51.39	1.58 (0.212)	**5.19 (0.026)**	1.91 (0.171)
DHEA-S (μg/mL)	1.75 (1.32–3.12)	1.27 (0.79–1.78)	1.00 (0.56–1.52)	0.43 (0.30–0.70)	**8.14 (0.006)**	**4.86 (0.031)**	0.04 (0.834)
Prolactin (ng/mL)	5.96 (4.69–7.73)	11.48 (7.38–15.82)	5.90 (4.48–8.94)	10.90 (7.30–12.86)	**5.40 (0.023)**	2.92 (0.092)	4.06 (0.055)
CRP (mg/L)	1.70 (0.90–3.80)	1.20 (0.90–2.10)	2.00 (1.00–3.40)	1.50 (0.70–2.10)	0.62 (0.435)	1.87 (0.175)	1.78 (0.675)

*DHEA-S, dehydroepiandrosterone-sulfate; HDL-C, high-density lipoprotein cholesterol; LDL-C, low-density lipoprotein cholesterol; PTSD, post-traumatic stress disorder; Q1–Q3, interquartile range; SD, standard deviation*.

*^a^Variables represented by median (Q1–Q3) were log-transformed*.

*^b^Statistically significant (*p* < 0.05) results are shown in bold*.

No significant main effects were recorded for serum lipids. However, since the interaction effect was significant for LDL-C, *post hoc* analyses showed significant elevation of LDL-C concentrations only in PTSD patients (*t* = 6.87, *p* < 0.001) and not in control subjects (*t* = 0.59, *p* = 0.559), although there were no group differences in individual time points. This tendency was also observed for triglycerides and total cholesterol as seen from raw values in Table 2. Tests of between-subject effects showed significant effect of smoking in RM ANCOVA model [*F*(1,75) = 11.07, *p* < 0.001] with triglycerides levels being significantly higher in smokers at the second assessment (*t* = 3.31, *p* = 0.001). Significant between-subjects effects in the model for total cholesterol were found for antipsychotics [*F*(1,75) = 5.57, *p* = 0.021] and hypolipidemics [*F*(1,75) = 4.62, *p* = 0.035]. Total cholesterol levels were higher in those using antipsychotics (*n* = 15) at the first assessment (*t* = 2.48, *p* = 0.022) and tended to be higher at the second assessment (*t* = 2.02, *p* = 0.057). As expected, total cholesterol levels were lower in patients using hypolipidemics (*n* = 7) at the first (*t* = 4.53, *p* = 0.001), as well as at the second assessment (*t* = 6.13, *p* < 0.001). No such effects were observed for LDL-C or HDL-C.

A significant effect of group was revealed for DHEA-S and prolactin, meaning that concentrations of these two hormones were altered in PTSD patients at both time points even after adjusting for confounding factors. DHEA-S levels were higher while prolactin levels were lower in PTSD patients. As shown by a significant effect of time, DHEA-S and cortisol levels declined in both groups.

C-reactive protein levels tended to be higher in PTSD patients at both time points. However, no significant effect in RM ANCOVA model was found. A significant interaction of time and smoking in tests of within-subjects effects [*F*(1,75) = 4.01, *p* = 0.049] indicated that smoking was an important confounding factor. Indeed, when RM ANOVA was performed without covariates, the effect of group was significant [*F*(1,75) = 5.33, *p* = 0.023]. Addition of smoking as covariate blunted the effect [*F*(1,75) = 3.04, *p* = 0.085]. Moreover, *t*-tests revealed higher CRP concentration in smokers (*n* = 38) compared to non-smokers (*n* = 52) (*t* = 2.28, *p* = 0.025).

### Multiplex analytes

The comparison of multiplex assay analytes between PTSD patients and controls at the first assessment is shown in Table 3. A significant difference was observed only for IL-8 with its concentration being lower in PTSD patients. When only drug-free patients (*n* = 12) were compared to control subjects (*n* = 32), IL-8 was still significantly lower in PTSD patients (*Z* = 2.54, *p* = 0.011) with no change in other variables. However, in one-way ANCOVA model with IL-8 as a dependent variable and age, smoking, alcohol use, and medication use (10 variables) as covariates, the effect of group was not significant [*F*(1,89) = 3.41, *p* = 0.069]. Concentrations of sCD40L and sPECAM-1 significantly declined in PTSD patients while levels of IL-8 and IL-1β were higher in comparison to levels at the first assessment (Table 4). A higher percentage of samples were detectable for IL-2 and IL-6 at the second assessment.

**Table 3:** **Comparison of multiplex assay analytes between PTSD patients and controls at the first assessment**.

Analyte	Assay sensitivity	PTSD (*n* = 69)		Controls (*n* = 32)		Statistical value^a^	*d*-value^b^	*p*-value^c^
		Median (Q1–Q3)	Detectable (%)	Median (Q1–Q3)	Detectable (%)	
sCD40L	0.02 ng/mL	4.72 (3.95–5.89)	69 (100)	4.51 (3.01–5.87)	32 (100)	1.02	0.027	0.313
sPECAM-1	0.8 ng/mL	229.76 (179.67–288.12)	69 (100)	191.52 (163.61–224.08)	32 (100)	1.15	0.025	0.253
sICAM-1	5.3 ng/mL	620.29 (484.78–854.38)	69 (100)	635.18 (468.07–917.76)	32 (100)	0.18	0.046	0.862
Leptin	0.05 ng/mL	17.97 (9.03–24.97)	69 (100)	15.52 (11.31–21.75)	32 (100)	−0.42	0.042	0.672
MIP-1α	1.0 pg/mL	31.28 (5.82–134.07)	55 (79)	48.25 (12.41–203.18)	27 (84)	−1.01	0.029	0.313
IL-8	0.5 pg/mL	39.50 (0.00–112.88)	45 (65)	184.58 (60.1–397.40)	29 (91)	−4.37	0.006	<0.001*
IL-1β	4.2 pg/mL	0.00 (0.00–34.47)	35 (51)	5.95 (0.00–17.34)	18 (56)	0.21	0.048	0.826
IL-2	16.4 pg/mL	0.00 (0.00–0.00)	15 (22)	0.00 (0.00–0.00)	4 (13)	Exact	0.033	0.412
TNF-α	3.2 pg/mL	0.00 (0.00–0.00)	14 (20)	0.00 (0.00–0.00)	4 (13)	Exact	0.035	0.413
IFN-γ	1.6 pg/mL	0.00 (0.00–0.00)	13 (19)	0.00 (0.00–0.00)	2 (6)	Exact	0.019	0.135
NGF	126.8 pg/mL	0.00 (0.00–0.00)	6 (9)	0.00 (0.00–0.00)	1 (3)	Exact	0.037	0.427
IL-4	20.8 pg/mL	0.00 (0.00–0.00)	4 (6)	0.00 (0.00–0.00)	1 (3)	Exact	0.038	0.491
IL-6	1.2 pg/mL	0.00 (0.00–0.00)	1 (1)	0.00 (0.00–0.00)	1 (3)	Exact	0.040	0.535

*IFN-γ, interferon-γ; IL, interleukin; MIP-1α, macrophage inflammatory protein-1α; NGF, nerve growth factor; PTSD, post-traumatic stress disorder; Q1–Q3, interquartile range; sCD40L, soluble CD40 ligand; SD, standard deviation; sICAM-1, soluble intercellular adhesion molecule-1; sPECAM-1, soluble platelet endothelial cell adhesion molecule-1; TNF-α, tumor necrosis factor-α*.

*^a^*t*-value: independent sample *t*-tests calculated for log-transformed variables (except IL-1β, *Z*-value: Mann–Whitney’s test), “Exact”: Fisher’s exact test*.

*^b^False discovery rate threshold value (*d*) calculated with significance level (α) set to 0.05 for 26 comparisons*.

^c^The difference is considered significant at α = 0.05 if *p*-value is less than the corresponding *d* value (labeled with *).

**Table 4:** **Comparison of multiplex assay analytes in PTSD patients (*n* = 47) between two assessments**.

Analyte	Median (Q1–Q3)		Detectable (%)		Statistical value^a^	*d*-value^b^	*p*-value^c^
	Time 1	Time 2	Time 1	Time 2	
sCD40L	4.76 (3.75–5.68)	4.26 (3.71–4.78)	47 (100)	47 (100)	−3.99	0.008	<0.001*
sPECAM-1	204.84 (166.08–286.69)	162.17 (120.78–213.94)	47 (100)	47 (100)	−5.82	0.002	<0.001*
sICAM-1	589.47 (470.83–787.31)	635.40 (468.53–1008.13)	47 (100)	47 (100)	1.32	0.021	0.194
Leptin	15.73 (8.41–26.67)	13.50 (0.66–23.18)	47 (100)	47 (100)	1.73	0.015	0.091
MIP-1α	30.28 (0.00–111.81)	32.33 (14.76–67.67)	35 (75)	43 (92)	1.20	0.023	0.236
IL-8	33.95 (0.00–135.12)	191.87 (55.22–405.51)	31 (66)	40 (85)	4.65	0.004	<0.001*
IL-1β	0.00 (0.00–34.47)	18.21 (0.00–31.51)	21 (44)	32 (68)	2.13	0.013	0.012*
IL-2	0.00 (0.00–0.00)	0.00 (0.00–100.53)	9 (19)	21 (45)	Exact	0.010	0.004*
TNF-α	0.00 (0.00–0.00)	0.00 (0.00–10.63)	10 (21)	12 (26)	Exact	0.044	0.791
IFN-γ	0.00 (0.00–0.00)	0.00 (0.00–0.00)	10 (21)	7 (15)	Exact	0.031	0.375
NGF	0.00 (0.00–0.00)	0.00 (0.00–0.00)	5 (11)	1 (2)	Exact	0.017	0.125
IL-4	0.00 (0.00–0.00)	0.00 (0.00–0.00)	4 (9)	4 (9)	Exact	0.050	0.999
IL-6	0.00 (0.00–0.00)	0.00 (0.00–0.00)	1 (2)	9 (19)	Exact	0.040	0.008*

*See Table 3 for abbreviations*.

*^a^*t*-value: dependent sample *t*-tests calculated for log-transformed variables (except IL-1β, *Z*-value: Wilcoxon signed-rank tests), “Exact”: McNemar’s exact test*.

*^b^False discovery rate threshold value (*d*) calculated with significance level (α) set to 0.05 for 26 comparisons*.

^c^The difference is considered significant at α = 0.05 if *p*-value is less than the corresponding *d* value (labeled with *).

Subgroup analyses revealed higher concentrations of sICAM-1 (Mann–Whitney *Z* = 3.26, *p* = 0.001) and leptin (*Z* = 2.84, *p* = 0.005) in patients taking hypolipidemics at the first assessment. At the second assessment, patients who were using hypolipidemics had higher concentrations of MIP-1α (*Z* = 2.83, *p* = 0.005). A lower concentration of sICAM-1 was observed in patients who were taking antipsychotics (*Z* = 2.87, *p* = 0.004) at the first assessment. No influence of smoking was revealed in subgroup analyses.

### Reproducibility and agreement between measurements

As seen in Table 5, fair to good reproducibility was observed for determination of all serum lipids, DHEA-S, CRP, sCD40L, and sICAM-1. Repeated measurements of prolactin, sPECAM, leptin, MIP-1α, and IL-1β showed excellent reproducibility. Determination of cortisol and IL-8 was associated with poor reproducibility meaning that within-subject variance was greater than between-subject variance. LASC 17-item PTSD index and STAI-Trait testing showed fair to good reproducibility while full scale LASC, BDI, and STAI-State were associated with poor reproducibility.

**Table 5:** **Intraclass correlation coefficient (ICC) and agreement between measurements**.

	ICC (95% CI) or kappa
Serum lipids
Triglycerides	0.56 (0.40–0.69)
Total cholesterol	0.69 (0.57–0.77)
LDL-C	0.63 (0.48–0.74)
HDL-C	0.73 (0.61–0.81)
Serum hormones
Cortisol	−0.17 (−0.37 to 0.03)
DHEA-S	0.50 (0.33–0.64)
Prolactin	0.79 (0.69–0.85)
CRP	0.41 (0.21–0.57)
Multiplex analytes
sCD40L	0.41 (0.14–0.63)
sPECAM-1	0.77 (0.62–0.86)
sICAM-1	0.59 (0.37–0.75)
Leptin	0.78 (0.63–0.87)
MIP-1α	0.85 (0.74–0.91)
IL-8	−0.08 (−0.36 to 0.21)
IL-1β	0.91 (0.85–0.95)
IL-2	0.271
TNF-α	0.171
IFN-γ	0.643
NGF	0.309
IL-4	0.453
IL-6	0.168
Psychometric tests
LASC 17-item PTSD index	0.45 (0.27–0.60)
LASC 43-item full scale index	0.39 (0.20–0.55)
BDI	0.06 (−0.15 to 0.26)
STAI-State	0.10 (−0.10 to 0.30)
STAI-Trait	0.45 (0.27 to 0.60)

*CI, confidence interval; for others, see Tables 1–4*.

Fair agreement between measurements was observed for IL-2 and NGF. Measurements of IL-4 and IFN-γ showed moderate and good agreement, respectively. Detection of TNF-α and IL-6 showed poor agreement between measurements.

### Correlations between biological variables and psychometric test scores

No correlation between psychometric test scores and biological variables in the group of PTSD patients was found to be significant after FDR correction for multiple correlations. However, we decided to report initially significant correlations as a guideline for future studies. Patients with detectable levels of IL-4 tended to score higher on LASC re-experiencing cluster of symptoms (ρ = 0.26, *p* = 0.032) at the first assessment. NGF levels above LOD were associated with higher severity of all three clusters of symptoms (re-experiencing: ρ = 0.24, *p* = 0.046; avoidance: ρ = 0.27, *p* = 0.024; arousal: ρ = 0.26, *p* = 0.028) at the first assessment time point. At the second assessment, avoidance symptoms were positively associated with sPECAM-1 concentration (ρ = 0.30, *p* = 0.044). Chemokine levels at the first assessments were positively correlated with arousal symptoms at the second assessment (MIP-1α, ρ = 0.29, *p* = 0.027; IL-8, ρ = 0.29, *p* = 0.027). Higher PTSD symptom severity at the first assessment was associated with lower cortisol levels at the second assessment (avoidance: ρ = −0.31, *p* = 0.035; 17-item PTSD index: ρ = 0.28, *p* = 0.026).

## Discussion

We found a robust association of combat-related chronic PTSD with higher serum levels of DHEA-S and lower levels of prolactin. The observed differences between PTSD patients and healthy controls persisted after 3 months. Repeated measurements of these markers were associated with good to excellent reproducibility. Cortisol levels were stable neither in PTSD patients nor in controls, and its determination was associated with poor reproducibility. Out of all cytokines and other immune-related markers measured, only IL-8 was significantly lower in PTSD patients in comparison to healthy controls. However, its concentration was not stable in PTSD patients as shown by a significant increase after 3 months and repeated measurements showed poor reproducibility. Although serum lipids did not differ between patients and controls in individual time points, a significant elevation of LDL-C was observed in PTSD patients.

The observed lack of difference in serum lipids is in line with a previous study of Croatian combat veterans with PTSD (35). In this study, patients were medicated and the authors argued that the combination of drugs might have affected final serum lipid levels although they did not control for medication in analyses. In the present study, we tried to control for medications that patients used and observed the opposite effect of atypical antipsychotics and hypolipidemics on total cholesterol levels, which was not surprising. Evidence supports efficacy of antipsychotics in treatment-resistant PTSD (63) and they are commonly used in treatment of combat veterans in Croatia (64). However, antipsychotics induce various metabolic alterations including dyslipidemia (65) so the use of hypolipidemics is justified. Previously studied samples of combat veterans with PTSD that exhibited elevated serum lipids did not include patients using antipsychotics (13, 15, 66) or lipid-lowering drugs (10). When we excluded the patients taking antipsychotics and hypolipidemics from analyses, the results remained the same, i.e., no difference in serum lipids at individual time points. However, we have observed a significant elevation in LDL-C after 3 months in PTSD patients but not in the control group. This tendency was observed for triglycerides and total cholesterol as well and it was even statistically significant when patients using antipsychotics and hypolipidemics were excluded from analyses (data not shown). Even if these findings are related to factors that were not controlled for, such as poor diet or lack of exercise, this information clearly shows that PTSD patients may be more prone to dyslipidemia, which is in line with recent findings of higher prevalence of metabolic syndrome in PTSD patients (17, 67).

Disrupted lipid metabolism may be associated with dysregulation of the HPA axis (19) but this was not evident from findings of basal morning cortisol levels in our study. We observed no difference between PTSD patients and controls and cortisol levels declined in both groups after 3 months. Low ICC (i.e., the poor reproducibility) indicated that cortisol determination was associated with low correlation between measurements, high between-subject, and low within-subject variance. This may be related to high biological variability of morning serum cortisol levels and/or measurement error associated with the method used to determine cortisol concentrations. We used enzyme immunoassay, which is comparable to radioimmunoassay (68), the most often used assay in PTSD-related basal cortisol studies. Both immunoassays exhibit cross-reactivity and specificity issues, especially if serum levels of cortisol are high (68) (in our study both PTSD patients and controls had cortisol levels around upper limits of the standard reference range). The use of gas chromatography–mass spectrometry may result in more reliable results (69). In addition, the observed decline in serum cortisol levels in both groups after 3 months may be due to seasonal variation (70), which must be taken into account as another source of variability in study design (duration of participant recruitment).

Although cortisol levels were not altered in PTSD patients, DHEA-S, which is also partly secreted from the adrenal gland in response to ACTH stimulation, was significantly higher in PTSD patients and repeated measurements were associated with good reproducibility. Circulatory DHEA-S levels were found to be elevated in chronic combat-related PTSD (27) and chronic PTSD with the history of childhood abuse (28, 43). Our study also partly replicated the findings from another longitudinal study (29) where PTSD patients tended to have higher DHEA-S levels across four time points separated by 3 months. However, authors did not provide information about the time span of participant recruitment and did not report significant changes over time. The significant decline of DHEA-S in both groups after 3 months observed in our study may be associated with seasonal variation as shown by Grade et al. (71) in healthy women (with lowest levels during summer and winter) even though a more recent study did not confirm seasonal variation of DHEA-S in either sex (72). Nevertheless, our results indicate that this source of variability should be considered in study design and that it may have contributed to negative findings of DHEA-S in PTSD (30, 40, 73). Robust results of elevated DHEA-S provide additional evidence of dysregulated HPA axis in chronic combat-related PTSD and highlight the importance of DHEA-S not only as a disease marker but also as a possible recovery marker (42) and, as shown recently, a susceptibility marker (74).

The finding of lower prolactin levels in PTSD patients may also be associated with dysregulation of the HPA axis. Previous studies showed enhanced prolactin suppression after a dexamethasone challenge in combat veterans with PTSD (38, 75) indicating that PTSD patients may be more prone to glucocorticoid-related modulation of factors involved in prolactin release. These factors could include serotonin since PTSD patients showed decreased prolactin response to serotonin-releasing agent (d-fenfluramine) compared to healthy controls (76). Although basal levels of prolactin were not altered in these studies, perhaps due to low sample sizes and lack of power, Olff et al. (40) found lower prolactin levels in chronic PTSD patients, which is consistent with our results. However, no changes in basal prolactin levels were also reported (39, 41) and one longitudinal study showed elevated prolactin levels in Croatian combat veterans with PTSD (24). The difference from the latter study may be because veterans were assessed earlier in the course of disease. Besides, elevated prolactin may have reflected residual effects of medication after a washout period (77). Considering medication, we expected to find higher prolactin levels in our sample of PTSD patients. Indeed, patients who were using antipsychotics tended to have higher levels of prolactin at the first assessment but this difference was not statistically significant. No significant association with other drugs was observed. Despite the use of prolactin-elevating medication, PTSD patients showed decreased prolactin levels that were stable after 3 months and its determination was associated with excellent reproducibility. This finding marks the importance of prolactin as a putative stable, although indirect, marker of enhanced HPA axis negative feedback system (78).

Despite known immuno-modulatory properties of DHEA-S (26) and prolactin (23), PTSD patients in our study did not show alterations in circulating levels of CRP or in pro-inflammatory cytokines (IL-1β, TNF-α, IL-6) in comparison to healthy controls. Cytokines associated with cell-mediated (Th1: IFN-γ, IL-2) and humoral (Th2: IL-4) immunity were not altered as well. Current findings in PTSD point to enhanced inflammation with disturbed Th1/Th2 balance (20, 79). There is also evidence of insufficient regulation of immune function as shown by reduction of regulatory T cells (Treg) in refugees with chronic PTSD (80). By analyzing Tregs in a random subset of PTSD patients from this study, we have found that they exhibit a different phenotype of Tregs that might be less suppressive (52). Although no difference in CRP and cytokines was observed between patients and controls at the first assessment, impaired immune regulation might be reflected in the significant rise and/or more patients having detectable levels of pro-inflammatory (IL-1β, IL-6) and Th1 (IL-2) cytokines after 3 months during winter when viral exposure was higher. Unfortunately, control subjects were not assessed at the second assessment so we could not test the hypothesis of higher susceptibility of PTSD patients to inflammatory responses. However, IL-1β determination was associated with excellent reproducibility. With previous evidence of elevated IL-1β in PTSD (81–83), further prospective studies examining the dynamics of this marker in PTSD are warranted. As for other pro-inflammatory cytokines (IL-6, TNF-α), the multiplex assay that we used was not sensitive enough to allow quantitative analyses. This was most evident for IL-6 with only one detectable sample at the first assessment. Low agreement between repeated detections of IL-6 and TNF-α suggests high variability over time with many samples that were negative at the first assessment being positive at the second and *vice versa*.

As for chemokines, in contrast to one previous study (22), MIP-1α was not elevated in PTSD patients but we found lower levels of IL-8, another chemokine that has already been found to be decreased in earthquake survivors with PTSD (84). This significance was attenuated after controlling for confounding factors and IL-8 levels were not stable, as seen by significant elevation after 3 months. Moreover, its measurements were associated with poor reproducibility which may in some extent explain different findings across studies (22, 45, 84). Interestingly, both chemokine levels at the first assessments were positively correlated with arousal symptoms after 3 months, which indicates possible predictive value of these markers.

An association of PTSD with higher levels of soluble adhesion molecules has been demonstrated in PTSD after myocardial infarction (85) and accidents (86) but a negative finding of sICAM-1 has also been reported (87). In the present study, neither concentrations of adhesion molecules (sICAM-1, sPECAM-1) nor levels of costimulatory molecule sCD40L were significantly altered in PTSD relative to healthy controls. These inflammatory markers are associated with endothelial dysfunction and platelet activation, playing a potential role as predictors of CVD (88). Von Känel et al. (89) found lower plasma sCD40L levels in patients with PTSD caused by myocardial infarction but only after controlling for depression symptoms, which positively correlated with sCD40L levels. PTSD patients in our study showed a significant decline in depression symptoms after 3 months, which may explain the observed decline in serum sCD40L levels. Since we have observed improvement of PTSD symptoms as well, in parallel with sPECAM-1 decline, adhesion molecules and sCD40L need further attention, possibly as therapy markers, especially because their determination was associated with good to excellent reproducibility. Moreover, sPECAM-1 tended to positively correlate with avoidance symptoms at the second assessment.

Given their important role in stress-related psychopathology (90, 91), it is surprising that leptin and NGF have not been more extensively studied in PTSD. One of the two leptin studies demonstrated elevated leptin in PTSD patients after myocardial infarction (89) and another in earthquake survivors with subsyndromal PTSD (92). We did not replicate these results and found no difference between PTSD patients and controls without changes in the PTSD group after 3 months. Leptin levels were affected by hypolipidemics but the difference between patients and controls remained non-significant even after patients using lipid-lowering drugs were excluded from analyses.

To our knowledge, soluble NGF has not been measured in PTSD. Unfortunately, the assay that we used was not sensitive enough to allow quantitative analyses. However, a small number of patients with detectable NGF at the first assessment tended to have more pronounced PTSD symptoms. Arousal or anxiety states are often accompanied by increases in NGF levels, which possibly reflect a central neuroprotective homeostatic mechanism with the potential to activate neuroendocrine and immune elements at the periphery (91). New evidence suggests that NGF can also be measured in saliva in response to stress (93). With the evidence of altered peripheral levels of other neurotrophins in PTSD (94, 95), future studies of NGF using more sensitive assays are warranted.

Although PTSD symptoms may fluctuate over time (96), we observed a significant decline in all three clusters of self-reported PTSD symptoms after 3 months. Croatian war veterans perceive extremely low levels of social acknowledgment (97) and the patients possibly saw the research initiative as a kind of organized care intervention especially since all veterans were gathered in the hospital in a single day for assessments. This itself might have had a therapeutic effect and resulted in better adherence to other forms of treatment, including medications. High motivation was also evident in a low dropout rate after 3 months. However, the observed changes in biological markers that were associated with PTSD (i.e., DHEA-S, prolactin) were parallel in both patients and controls. On the other hand, the possibility remains that the changes observed in markers that were not measured in controls at the second assessment were associated with the improvement of symptoms. As mentioned before, the observed decline in sCD40L and sPECAM-1 needs further attention with better assessment of PTSD symptoms and in relation to specific therapeutic procedures.

### Strength and limitations

One of the limitations of the study is that we did not administer structured interviews for assessment of the symptoms. The main reason for using self-report scales was time constraint, since we wanted to perform all assessments in a single day to minimize sources of variability. PTSD patients comprised a very homogeneous group of severely traumatized combat veterans who started their treatment shortly after the war in Croatia. Since then, they have participated in various treatment programs and they have been assessed with various instruments on multiple occasions according to multimodal assessment principles (98), including Clinician-Administered PTSD Scale (CAPS) (99). Furthermore, we excluded patients who did not satisfy criteria for PTSD according to LASC diagnostic items. Besides, repeated assessments of LASC 17-item PTSD index showed good reproducibility, which adds to the reliability of diagnosis. Another caveat is that we did not specifically control for possible comorbid depression although BDI scores were relatively high on the day of the first assessment. However, while some biological measures tended to correlate with severity of PTSD symptoms, no correlation was found with BDI scores at either assessment. Moreover, BDI scores at the second assessment indicated mild depression at most. The use of medication is another limitation, which could not be avoided due to ethical reasons. We tried to control for drugs that patients used in statistical analyses. The use of antipsychotics and hypolipidemics had a significant effect on several variables but we decided not to exclude those patients to preserve power. Analyses of multiplex analytes could not be modeled entirely to include covariates so we excluded patients using those drugs and the results for affected analytes remained similar (sICAM-1, MIP-1α, and leptin). Finally, the study did not have available body mass index (BMI) data. In our previous study on another sample of PTSD patients from the same area (meaning similar lifestyle and dietary habits), we found no difference between PTSD patients and healthy controls regarding BMI (100). Both groups were slightly overweight in line with the study encompassing a larger sample of Croatian war veterans with PTSD and without PTSD in comparison to a nation-wide civilian sample (101). Considering all this, we presume that BMI did not have large effect on the comparison of variables known to be affected by BMI.

The main strength of the current study is that we measured a wide array of biological variables in a relatively large group of PTSD patients and repeated the measurements after a reasonable period to assess how stable observed changes are over time. All participants were assessed in a single day at each assessment time point and blood was drawn in the morning during a limited period, thus minimizing circadian and eliminating monthly and seasonal variations in biological variables. The same group of professionals performed assessments at both time points using the same methods. This allowed us to compare intra- and inter-individual variability and provide reliability estimates as a guideline for future studies. The assessment of the control group at both time points enabled us to test if the changes over time in a number of variables were specifically related to PTSD.

## Conclusion

Although many biological alterations have been reported in PTSD, no reliable and specific PTSD biomarker has been identified to date (46). Differences in findings across studies may be explained by variations in sample sizes, sample characteristics (e.g., type of trauma, time elapsed since trauma, comorbidities), and large inter-individual variability of biological variables. One of the requirements that PTSD biomarkers should fulfill is reliability, i.e., repeated testing should produce similar results. The results of our repeated assessments indicate that some biological changes may persist over a longer period during the course of chronic PTSD in treatment-resistant war veterans and have potential to be reliable markers of disease (e.g., DHEA-S and prolactin). Other alterations were not so constant, such as serum lipids and cytokines, but the observed changes probably did reflect differences in reactivity of neuroendocrine-immune networks. Regardless of which direction the change takes, disrupted homeostasis may eventually lead to disease. Given the seasonal variations of some variables and high intra-individual variability, cross-sectional studies should be carefully designed with participant recruitment over a very limited time period. Additionally, this emphasizes the importance of longitudinal studies in investigations of PTSD biomarkers.

## Author Contributions

SR, AS, NA, MJ, and AV conceived and designed the study. MJ, KB, AM, and VV performed laboratory experiments. NA organized and supervised psychological testing and blood sampling. AV and AS performed statistical analyses of the data. TJ contributed to statistical analyses and interpretation of the data. AV and MJ wrote the first draft of the manuscript. KB, AM, VV, NA, SR, TJ, and AS critically revised the manuscript. All authors read and approved the final manuscript.

## Conflict of Interest Statement

The authors declare that the research was conducted in the absence of any commercial or financial relationships that could be construed as a potential conflict of interest.
